# Bibliometric and visual analysis of time-restricted eating

**DOI:** 10.3389/fnut.2022.979702

**Published:** 2022-08-09

**Authors:** Shuai Wang, Xiaoxiao Lin, Yihong Guan, Jinyu Huang

**Affiliations:** ^1^Department of Translation Medicine Center, Affiliated Hangzhou First People’s Hospital, Zhejiang University School of Medicine, Hangzhou, China; ^2^Department of Cardiology, Affiliated Hangzhou First People’s Hospital, Zhejiang University School of Medicine, Hangzhou, China; ^3^The Fourth School of Clinical Medicine, Zhejiang Chinese Medical University, Hangzhou, China

**Keywords:** bibliometric analysis, visual maps, time-restricted eating (TRE), time-restricted feeding (TRF), obesity

## Abstract

An increasing number of studies have shown the effects of time-restricted eating (TRE) on metabolic diseases and cardiovascular diseases associated with obesity. However, no bibliometric analyses were conducted in this field systematically. In our study, we aimed to visualize the publications about TRE to determine the frontiers and hotspots and then provide references and guidance for further studies. Publications about TRE were exported from the Web of Science Core Collection (WOSCC) database. VOSviewer 1.6.16 was adopted to perform the bibliometric analysis. In our study, a total of 414 publications with 298 articles and 116 reviews were included. The publications in this field showed an upward trend from 2016. A total of 2016 authors contributed to this field. The most productive authors were Satchidananda Panda, Krista A Varady and Emily NC Manoogian. All publications were distributed from about 624 organizations from 49 Countries/Regions. The leading institutions were the Salk Institute for Biological Studies, the University of California San Diego and the University of Alabama at Birmingham, and the most productive countries were the United States, the People’s Republic of China and Japan. All publications were from 182 journals, and the most productive journals were Nutrients, Frontiers in Nutrition and Cell Metabolism. The first highest cited reference with 991 citations was published in Cell Metabolism, and authored by Satchidananda Panda et al. There were four indicating research directions, and the keywords of the green cluster were time-restricted feeding, metabolism, circadian clock, and circadian rhythm. The keywords of the blue cluster were obesity, health, diet, and food intake. The keywords of the red cluster were intermittent fasting, weight loss, caloric restriction, and time-restricted eating. The keywords of the yellow cluster were insulin resistance, metabolic disease, cardiovascular disease, and caloric intake. The main research hotspots in the TRE field were TRE and circadian rhythm, TRE and obesity, TRE and metabolic disease, and TRE and cardiovascular disease. TRE represents new directions to evaluate the effects of the timing of eating on different diseases, especially obesity, Type 2 diabetes mellitus and cardiovascular disease. Previous studies have generated impressive data on the effects of TRE on metabolic diseases and cardiovascular diseases associated with obesity. More high-quality studies are needed to assess the mechanism and efficacy of TRE in a wide range of populations and diseases.

## Introduction

Time-restricted eating (TRE) was an approach that all calorie intake is restricted within 12 h without reducing calories (1–5). It was an emerging behavioral intervention which was based on the circadian rhythms in metabolism and physiology (6–9). TRE has been proven to attenuate or prevent the severity of some metabolic diseases including dyslipidemia, hepatic steatosis, obesity, and age-related decline in cardiac function in preclinical animal models (1). TRE could reduce glucose intolerance, body weight, dyslipidemia, and hypertension in pilot human studies. TRF had pleiotropic effects on gut microbiome composition and multiple pathways in different organs (1). In order to assess TRE benefits accurately, it was necessary to develop new methods to promote and monitor compliance with a daily eating patterns in humans.

An increasing number of studies have shown the effects of TRE on metabolic diseases and cardiovascular disease associated with obesity. However, these findings were not summarized and analyzed as a whole. Bibliometric analysis was widely used to investigate the developmental trends and main findings *via* quantitative analysis of previous scientific literature. In our study, we conducted a bibliometric analysis of TRE to display the research status and draw visual maps and explored trends and hotspots in this field to provide the reference to future studies.

## Materials and methods

### Search strategy

The Web of Science Core Collection (WoSCC) is widely used for bibliometric analysis, which is the most authoritative citation-based database with powerful indexing functions (10, 11). Therefore, literature related to TRE was downloaded from the WoSCC. For TRE, there are two forms of expression including time restricted feeding and time restricted eating, so the search term was TS = “time restricted feeding” or “time restricted eating” or “time-restricted feeding” or “time-restricted eating.” The search results were confined by types of articles and reviews, publication data from inception to May 1, 2022, and language of English.

### Data collection and analysis

The relevant records with “Full Record and Cited References” were exported in the format of “Plain Text.” The resulting set of publications was analyzed bibliometrically. The top 10 high-yield authors, institutions, country/region and journals according to publications and the characteristics of the top 20 highest cited publications were summarized.

VOSviewer is a bibliometric tool which is developed for constructing and viewing bibliometric maps (12, 13). The distance and color represent these nodes are distributed in two-dimensional spaces (associations and the times). The larger its label and its circle when the more important an item. In our study, VOSviewer 1.6.16 software was used to perform co-authorship of a complete list of authors, organizations, countries, citations of journal and publications, and co-occurrence of all keywords. The minimum number set for the network was five.

## Results

### Publication output and temporal trend

**FIGURE 1:**
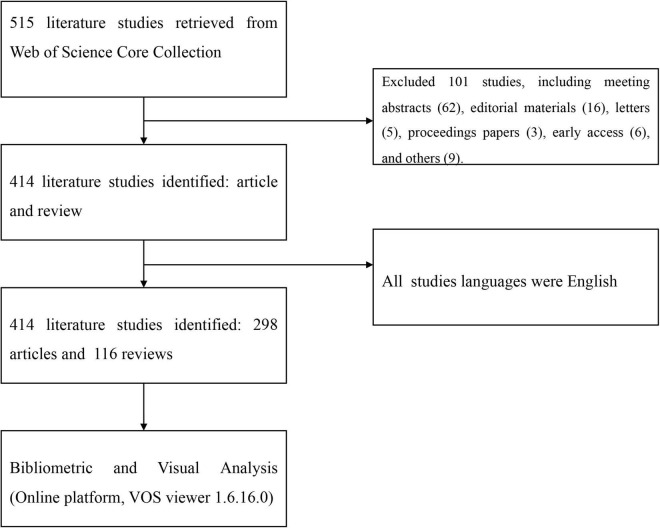
Flowchart of the inclusion and exclusion criteria.

**FIGURE 2:**
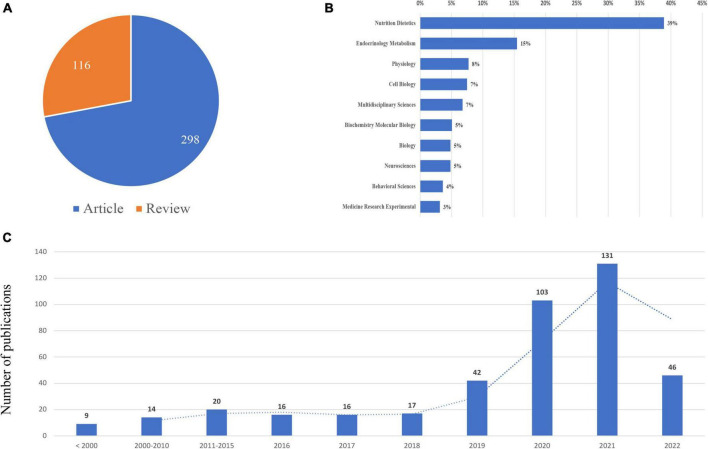
Yearly quantity and literature type of publications on time-restricted eating (TRE) from inception to May 1, 2022. **(A)** Literature types distribution. **(B)** Subject categories distribution. **(C)** Annual publications quantitative distribution.

A total of 414 publications (298 articles and 116 reviews) were included, which was shown in Figure 1. The Publications in this field of TRE showed an upward trend from 2016 sharply. The top subject categories in the analyzed publications were nutrition dietetics (161 publications) and endocrinology metabolism (64 publications), which was shown in Figure 2.

### Distribution of authors

**FIGURE 3:**
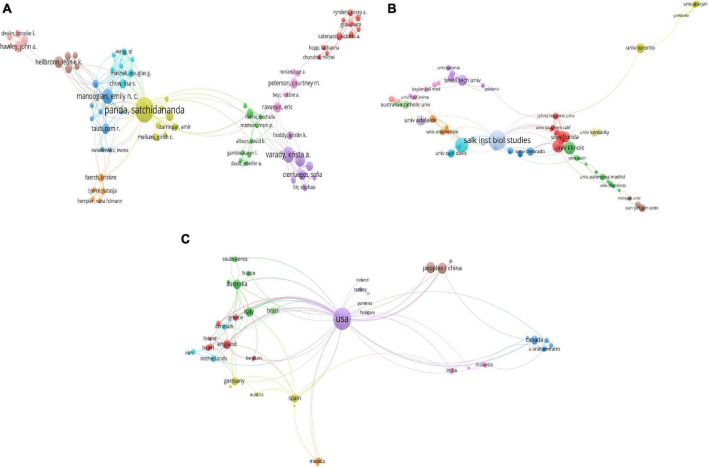
Visualization knowledge maps of the co-authorship. **(A)** The co-authorship map of authors which indicates the authors that cooperate in the field of time-restricted eating (TRE); **(B)** the co-authorship map of organizations. Salk Institute for Biological Studies has published the most related papers (24 items); **(C)** the co-authorship map of countries. The number of collaborators with United States is 184 and the total link strength is 114. Different colors indicate different clusters and the size of nodes indicates the number of publications. The thickness of the lines represents the link strength of the countries.

A total of 2016 authors contributed to this field. Among them, Satchidananda Panda was the most productive author from the United States with 26 publications and an H-index of 15, followed by Krista A Varady with 12 publications and an H-index of 8, Emily NC Manoogian with 11 publications and an H-index of 5, Kelsey Gabel with nine publications and an H-index of 5 and Grant M Tinsley with 8 publications and an H-index of 6. Satchidananda Panda, Krista A Varady and Emily N C Manoogian were the most frequently cited authors. The co-authorship map of the authors was shown in the Figure 3A.

### Distribution by country/region and institution

All publications were distributed from about 624 organizations from 49 Countries/Regions. The leading institutions were the Salk Institute for Biological Studies (24 publications with 4,003 citations), University of California San Diego (16 Publications with 2,644 citations), the University of Alabama at Birmingham (13 publications with 1,088 citations), University of Illinois (13 publications with 617 citations) and Texas Tech University (10 publications with 609 citations). The network visualization map of institutions was shown in Figure 3B. For Country/Region, the United States had the most publications with 184 documents, followed by the People’s Republic of China (33 documents), Japan (32 documents), Australia (24 documents) and Italy (21 documents). The network visualization map of the Country/Region was shown in Figure 3C. The top 10 high-yield authors, institutions and country/regions according to publications were summarized in Table 1.

**TABLE 1:** Ranking of the top 10 authors, institutions, and countries.

Items	Publications				
	Ranking	Country	Number	Citations	H-index
Country	1	United States	184	8,117	41
	2	People’s Republic of China	33	282	9
	3	Japan	32	611	14
	4	Australia	24	420	11
	5	Italy	21	1,492	13
	6	England	20	874	9
	7	Canada	19	185	7
	8	Spain	17	245	7
	9	Germany	16	132	6
	10	Brazil	14	327	7
Institution	1	Salk Institute for Biological Studies	24	4,003	15
	2	University of California San Diego	16	2,644	10
	3	University of Alabama at Birmingham	13	1,088	8
	4	University of Illinois	13	617	9
	5	Texas Tech University	10	609	7
	6	Pennington Biomedical Research Center	10	1,275	8
	7	University of Florida	10	383	5
	8	University of Adelaide	10	272	7
	9	University of Toronto	9	56	3
	10	Australian Catholic University	7	141	5
Author	1	Satchidananda Panda	26	4,018	15
	2	Krista A Varady	12	571	8
	3	Emily N C Manoogian	11	556	5
	4	Kelsey Gabel	9	171	5
	5	Grant M Tinsley	8	377	6
	6	Dandan Hu	7	58	5
	7	Leonie K Heilbronn	7	245	5
	8	John A Hawley	7	141	5
	9	Sofia Cienfuegos	6	124	3
	10	Yilei Mao	6	51	4

### Distribution by journal

**FIGURE 4:**
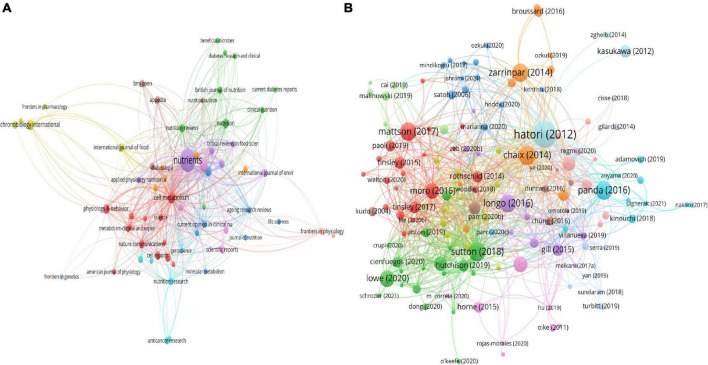
Visualization knowledge maps of citation. **(A)** Citation of Journal; **(B)** citation of documents. Different color indicates different clusters. The size of the nodes represents the counts of citations. The distance between the two nodes indicates their correlation.

All publications were from 182 journals. Among them, Nutrients was the most productive journal with 65 documents and 972 citations, which was followed by Frontiers in Nutrition with 13 documents and 101 citations, and Cell Metabolism with 10 documents and 3,224 citations. The most frequently cited journal was Cell Metabolism. The network visualization map of all journals was shown in Figure 4A. The top 10 high-yield according to publications were summarized in Table 2.

**TABLE 2:** Ranking of the top 10 journals based on publications.

Ranking	Journal name	Country	Counts	Citation	H-index
1	Nutrients	Switzerland	65	972	16
2	Frontiers in Nutrition	Switzerland	13	101	6
3	Cell Metabolism	United States	10	3,224	10
4	Chronobiology International	United States	9	149	5
5	Obesity	United States	8	319	5
6	Nutrition	United States	7	40	3
7	Physiology & Behavior	United States	7	115	4
8	Advances in Nutrition	United States	5	11	1
9	American Journal of Clinical Nutrition	United States	5	195	3
10	Nutrition Reviews	United States	5	313	4

### Analysis of high-cited documents

The characteristics of the top 20 highest cited publications were summarized in Table 3 (6, 14–32). The top three high cited articles were as follows: the first article with 991 citations was published in Cell Metabolism and authored by Hatori et al. (16). In this article, they found that mice with TRF were protected against hyperinsulinemia, inflammation, hepatic steatosis and obesity. The TRF regimen could improve oscillations of the circadian clock and target genes’ expression and mTOR, CREB, and AMPK pathway function. They demonstrated that the TRF regimen was a non-pharmacological approach against obesity and associated diseases in mice. The second article with 442 citations was published in Cell Metabolism and authored by Chaix et al. (14). In this article, they tested TRF in mice under diverse nutritional challenges and found that TRE could attenuate metabolic diseases from different obesogenic diets, and the protective effects existed even if the TRF was temporarily interrupted. TRF could stabilize and reverse the progression of metabolic diseases in mice with preexisting type II diabetes and obesity. They established clinically relevant parameters of TRF and believed that TRE could prevent and treat obesity and metabolic disorders including hepatic steatosis, hypercholesterolemia and type II diabetes. The third article with 417 citations was published in Cell Metabolism and authored by Sutton et al. (27), and it is the first trial of early TRE. In this study, they could improve appetite, oxidative stress, cell responsiveness, blood pressure, and insulin sensitivity in men with prediabetes. They showed that early TRF (eTRF) improves some aspects of cardiometabolic health in humans for the first time and these effects were not solely due to weight loss. The network visualization map of cited documents was shown in Figure 4B.

**TABLE 3:** Ranking of the top 20 highest cited references.

Rank	Title	Journal	Total citations	References
1	Time-restricted feeding without reducing caloric intake prevents metabolic diseases in mice fed a high-fat diet.	Cell Metabolism	991	Hatori et al. (16)
2	Time-restricted feeding is a preventative and therapeutic intervention against diverse nutritional challenges.	Cell Metabolism	442	Chaix et al. (8)
3	Early time-restricted feeding improves insulin sensitivity, blood pressure, and oxidative stress even without weight loss in men with prediabetes.	Cell Metabolism	417	Sutton et al. (27)
4	Circadian physiology of metabolism.	Science	394	Panda (25)
5	Diet and feeding pattern affect the diurnal dynamics of the gut microbiome.	Cell Metabolism	386	Zarrinpar et al. (32)
6	Impact of intermittent fasting on health and disease processes.	Ageing Research Reviews	375	Mattson et al. (23)
7	Fasting, circadian rhythms, and time-restricted feeding in healthy lifespan.	Cell Metabolism	372	Longo and Panda (19)
8	Effects of time-restricted eating on weight loss and other metabolic parameters in women and men with overweight and obesity the TREAT randomized clinical trial.	JAMA Internal Medicine	291	Lowe et al. (20)
9	Meal frequency and timing in health and disease.	Proceedings of the National Academy of Sciences of The United States of America	272	Mattson et al. (22)
10	Effects of eight weeks of time-restricted feeding (16/8) on basal metabolism, maximal strength, body composition, inflammation, and cardiovascular risk factors in resistance-trained males.	Journal of Translational Medicine	250	Moro et al. (24)
11	Ten-hour time-restricted eating reduces weight, blood pressure, and atherogenic lipids in patients with metabolic syndrome.	Cell Metabolism	214	Wilkinson et al. (30)
12	Time-restricted feeding prevents obesity and metabolic syndrome in mice lacking a circadian clock.	Cell Metabolism	214	Chaix et al. (6)
13	Early time-restricted feeding improves 24-hour glucose levels and affects markers of the circadian clock, aging, and autophagy in humans.	Nutrients	162	Jamshed et al. (17)
14	Human blood metabolite timetable indicates internal body time.	Proceedings of the National Academy of Sciences of The United States of America	147	Kasukawa et al. (18)
15	Time-restricted feeding attenuates age-related cardiac decline in Drosophila.	Science	144	Gill et al. (15)
16	Time-restricted feeding in young men performing resistance training: A randomized controlled trial.	European Journal of Sport Science	130	Tinsley et al. (28)
17	Effects of intermittent fasting on body composition and clinical health markers in humans.	Nutrition Reviews	129	Tinsley and La Bounty (29)
18	Daily eating patterns and their impact on health and disease.	Trends in Endocrinology and Metabolism	123	Zarrinpar et al. (31)
19	Circadian rhythms, time-restricted feeding, and healthy aging.	Ageing Research Reviews	122	Manoogian and Panda (21)
20	Time-restricted feeding and risk of metabolic disease: A review of human and animal studies.	Nutrition Reviews	122	Rothschild et al. (46)

### Analysis of all keywords co-occurrence clusters

**FIGURE 5:**
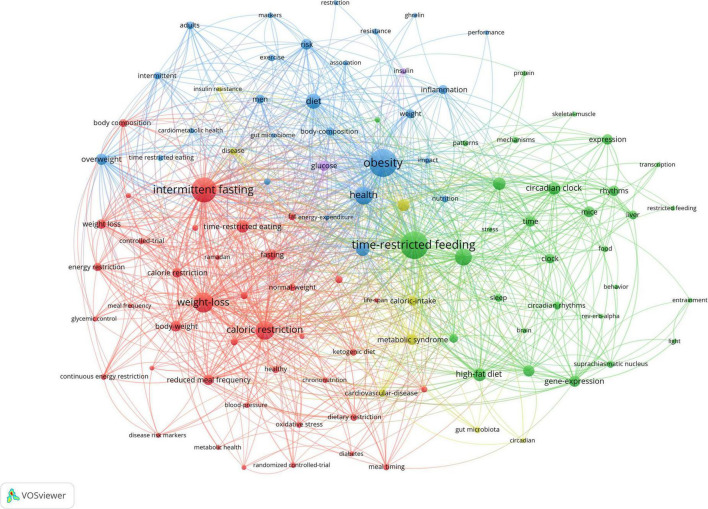
Visualization of keyword co-occurrence analysis. The size of nodes indicates the frequency of occurrences of the keywords. The lines between the nodes represents their co-occurrence in the same publication. The shorter the distance between two nodes, the larger the number of co-occurrence of the two keywords.

The network visualization map of keywords was shown in Figure 5, and the clusters of red, green, yellow and blue, including four indicating research directions. The keywords of the green cluster were time-restricted feeding, metabolism, circadian clock, and circadian rhythm. The keywords of the blue cluster were obesity, health, diet, and food intake. The keywords of the red cluster were intermittent fasting, weight loss, caloric restriction, and time-restricted eating. The keywords of the yellow cluster were insulin resistance, metabolic disease, cardiovascular disease, and caloric intake.

## Discussion

### General information

To our best knowledge, this is the first study of bibliometric and visual analysis for TRE. In our study, a total of 414 publications with 298 articles and 116 reviews were included. The publications in this field showed an upward trend from 2016. A total of 2016 authors contributed to this field.

The most productive authors were Satchidananda Panda, Krista A Varady, and Emily NC Manoogian. All publications were distributed from about 624 organizations from 49 Countries/Regions. The leading institutions were the Salk Institute for Biological Studies, the University of California San Diego and the University of Alabama at Birmingham, and the most productive countries were the United States, the People’s Republic of China and Japan. All publications were from 182 journals, and the most productive journals were Nutrients, Frontiers in Nutrition and Cell Metabolism. The first highest cited reference with 991 citations was published in Cell Metabolism, and authored by Satchidananda Panda et al. There were four indicating research directions, and the keywords of the green cluster were time-restricted feeding, metabolism, circadian clock, and circadian rhythm. The keywords of the blue cluster were obesity, health, diet, and food intake. The keywords of the red cluster were intermittent fasting, weight loss, caloric restriction, and time-restricted eating. The keywords of the yellow cluster were insulin resistance, metabolic disease, cardiovascular disease, and caloric intake.

In this bibliometric analysis, most of the articles were published from the United States, the People’s Republic of China and Japan, and the percentage of articles from the United States was 44%(184/414). Of the top 10 institutions, seven were from the United States. Of the top 10 productive author, six were from the United States. In the field of TRE, the prominent contribution was from the United States. It indicates that the studies from other countries need to be enhanced.

### Hotspots and frontiers

Through the combination of highest cited references and analysis of all keywords co-occurrence clusters, the research hotspots and frontiers were as follows: (1) TRE and circadian rhythm. In the top 20 highest cited references, five explored the TRE and the circadian rhythm, and the keywords of circadian clock and circadian were in the green cluster (6, 17, 19, 21, 25). The rhythm internal circadian rhythms play an important role in physiology and overall health (21). Disrupted circadian rhythms would compromise health and increase the risk of disease. Erratic lifestyle including jet-lag, shift work and aberrant sleep and eating patterns contribute to circadian rhythm disruption, which compromises multiple levels of physiology for inflammation and metabolism, and increases the risk for cardiovascular disease, obesity and metabolic disease. Researches in rodents have demonstrated that TRE could restore robust circadian rhythms and improve health, and decrease the risk of disease to extend healthspan. (2) TRE and obesity. In the top 20 highest cited references, four explored the TRE and obesity, and the keyword of obesity was in the blue cluster (6, 14, 20, 29). Cienfuegos et al. (33) showed that 6 and 4-h TRF could induce mild reductions in body weight and may be promising interventions for weight loss. Lowe et al. (20) demonstrated there was a significant weight loss in the TRE group but not in e consistent meal timing (CMT) group. A systematic review and meta-analysis showed that the TRF regimen had a significant weight loss compared with the unrestricted time regimen, which was mainly attributed to the loss of lean mass, not fat mass (20, 33–39). Recently, Liu et al. (40) showed that, compared with calorie restriction (CR), TRE was not more beneficial in the reduction of body weight and body fat. Thomas et al. (41) demonstrated that early TRE plus daily CR (E-TRE + DCR) resulted in similar levels of weight loss compared with DCR alone, and may be an acceptable dietary strategy. A recent study (42) has demonstrated that 30%-CR could extend lifespan by 10% while TRE and CR act together extended lifespan by 35% in male C57BL/6J mice, and this effect was independent of body weight. Although a regimen of TRE was not more beneficial for the reduction in body weight compared with CR, the effect of TRE on other factors such as longevity should be explored in further studies. (3) TRE and metabolic disease. In the top 20 highest cited references, six explored TRE and metabolic disease, and the keywords of metabolic disease and insulin resistance were in the yellow cluster (6, 16, 24, 27, 30, 43). Wilkinson et al. (30) demonstrated that TRE could improve the cardiometabolic health of patients with metabolic syndrome and could be added to standard medical practice. Sutton et al. (27) did the first study to prove that eTRF could improve some aspects of cardiometabolic health which were not solely due to weight loss. In their study, they found that eTRF could improve appetite, oxidative stress, cell responsiveness, blood pressure, and insulin sensitivity in men with prediabetes. (4) TRE and cardiovascular disease. In the top 20 highest cited references, three explored TRE and cardiovascular disease, and the keyword of cardiovascular disease was in the yellow cluster (15, 24, 27). Several trails (24, 27, 43–45) have evaluated the effect of TRE on parameters of cardiovascular health including plasma lipid concentrations and blood pressure. Wilkinson et al reported that TRF led to 12% reductions for LDL cholesterol levels in subjects with metabolic syndrome. Two studies reported minor decreases in triglyceride levels. Some studies reported decreases in diastolic (7–9%) and systolic (4–9%) blood pressure.

### Strengths and limitations

This was the first study to conduct a bibliometric analysis of TRE. In our study, we displayed the research status and draw visual maps, and explored trends and hotspots in this field to provide the reference for future studies. There were several limitations in our study. First, all data were extracted from the WoSCC, and other databases such as Medline and Embase were not used. Second, only studies published in English were included. Finally, bias may still exist despite our normalization procedures. Further studies will provide new insight into this field.

## Conclusion

The main research hotspots in the TRE field were TRE and the circadian rhythm, TRE and obesity, TRE and metabolic disease, and TRE and cardiovascular disease. TRE represents new directions to evaluate the effects of the timing of eating on different diseases, especially for obesity, Type 2 diabetes mellitus and cardiovascular disease. Previous studies have generated impressive data on the effects of TRE on metabolic diseases and cardiovascular diseases associated with obesity. More high-quality studies are needed to assess the mechanism and efficacy of TRE in a wide range of populations and diseases.

## Data availability statement

The original contributions presented in this study are included in the article/supplementary material, further inquiries can be directed to the corresponding authors.

## Author contributions

SW, XL, and YG were responsible for data collection, investigation, figures and tables construction, and writing the original draft. JH contributed to the discussion and final review and editing. All authors reviewed and edited the final manuscript.
